# Four Decades of Carrier Detection and Prenatal Diagnosis in Hemophilia A: Historical Overview, State of the Art and Future Directions

**DOI:** 10.3390/ijms241411846

**Published:** 2023-07-24

**Authors:** Rima Dardik, Szymon Janczar, Shadan Lalezari, Einat Avishai, Sarina Levy-Mendelovich, Assaf Arie Barg, Uri Martinowitz, Katarzyna Babol-Pokora, Wojciech Mlynarski, Gili Kenet

**Affiliations:** 1 National Hemophilia Center, Sheba Medical Center, Ramat Gan 52621, Israel; shadan.lalezari@sheba.health.gov.il (S.L.); einat.avishai@sheba.health.gov.il (E.A.); sarina.levy@sheba.health.gov.il (S.L.-M.); assaf.barg@sheba.health.gov.il (A.A.B.); martinur@bezeqint.net (U.M.); gili.kenet@sheba.health.gov.il (G.K.); 2 Amalia Biron Research Institute of Thrombosis and Hemostasis, Sackler School of Medicine, Tel Aviv University, Tel Aviv 52621, Israel; 3 Department of Pediatrics, Oncology and Hematology, Medical University of Lodz, 90-419 Lodz, Poland; szymon.janczar@umed.lodz.pl (S.J.); katarzyna.babol-pokora@umed.lodz.pl (K.B.-P.); wojciech.mlynarski@umed.lodz.pl (W.M.)

**Keywords:** hemophilia A, factor VIII, *F8* gene, carrier detection, prenatal diagnosis, preimplantation genetic diagnosis, X chromosome inactivation, whole-exome sequencing

## Abstract

Hemophilia A (HA), a rare recessive X-linked bleeding disorder, is caused by either deficiency or dysfunction of coagulation factor VIII (FVIII) resulting from deleterious mutations in the F8 gene encoding FVIII. Over the last 4 decades, the methods aimed at determining the HA carrier status in female relatives of HA patients have evolved from phenotypic studies based on coagulation tests providing merely probabilistic results, via genetic linkage studies based on polymorphic markers providing more accurate results, to next generation sequencing studies enabling highly precise identification of the causative F8 mutation. In parallel, the options for prenatal diagnosis of HA have progressed from examination of FVIII levels in fetal blood samples at weeks 20–22 of pregnancy to genetic analysis of fetal DNA extracted from chorionic villus tissue at weeks 11–14 of pregnancy. In some countries, in vitro fertilization (IVF) combined with preimplantation genetic diagnosis (PGD) has gradually become the procedure of choice for HA carriers who wish to prevent further transmission of HA without the need to undergo termination of pregnancies diagnosed with affected fetuses. In rare cases, genetic analysis of a HA carrier might be complicated by skewed X chromosome inactivation (XCI) of her non-hemophilic X chromosome, thus leading to the phenotypic manifestation of moderate to severe HA. Such skewed XCI may be associated with deleterious mutations in X-linked genes located on the non-hemophilic X chromosome, which should be considered in the process of genetic counseling and PGD planning for the symptomatic HA carrier. Therefore, whole exome sequencing, combined with X-chromosome targeted bioinformatic analysis, is highly recommended for symptomatic HA carriers diagnosed with skewed XCI in order to identify additional deleterious mutations potentially involved in XCI skewing. Identification of such mutations, which may profoundly impact the reproductive choices of HA carriers with skewed XCI, is extremely important.

## 1. Introduction

Hemophilia A (HA) is a rare bleeding disorder resulting from either deficiency or dysfunction of coagulation factor VIII (FVIII), caused by deleterious mutations in the F8 gene encoding FVIII (NM_000132.3). The F8 gene is located at the end of the long arm of the X chromosome in Xq28. Therefore, HA is a recessive X-linked disorder manifested predominantly by hemizygous male patients and transmitted by heterozygous female carriers.

Over the last 4 decades, genetic analyses of HA families aimed at assessing the carrier status of potential carriers have gradually evolved from the construction of pedigree charts combined with phenotypic studies [1,2], yielding merely probabilistic data, to more accurate genetic linkage studies based on polymorphic markers [3,4]. With the development of sequencing techniques, linkage studies have been replaced by direct sequencing and next-generation sequencing (NGS) studies, enabling precise identification of the causative *F8* mutation in most cases. In parallel, the options for prenatal diagnosis of HA have progressed from fetal blood sampling providing diagnostic results at weeks 20–22 of pregnancy [4,5] to genetic analysis of fetal DNA extracted from chorionic villus tissue at weeks 11–14 of pregnancy [4]. The development of preimplantation genetic diagnosis (PGD) enables hemophilia carriers to prevent disease transmission to their offspring while avoiding the psychological, religious, and cultural challenges that may be associated with the termination of pregnancies diagnosed with affected fetuses [6].

**Figure 1:**
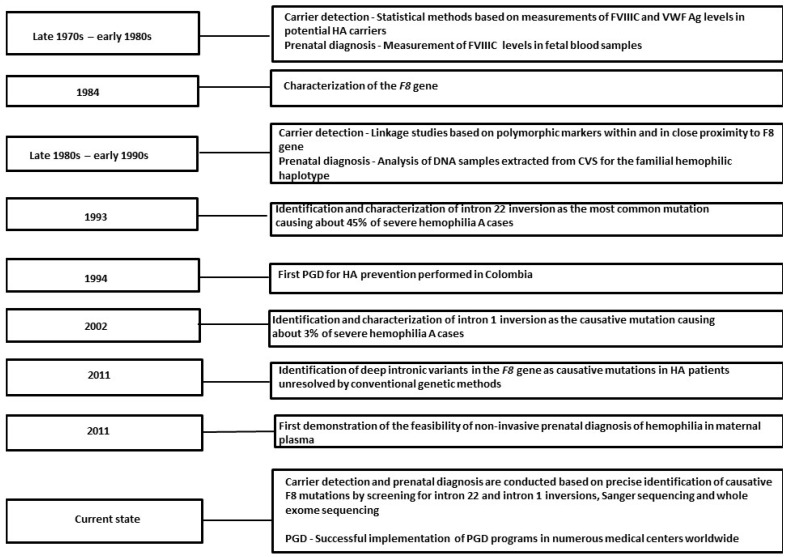
Timeline of carrier detection and prenatal diagnosis in hemophilia A. Abbreviations: FVIIIC: Factor VIII coagulant activity; HA: Hemophilia A; PGD: Preimplantation genetic diagnosis; VWF: von Willebrand factor.

In this review, we provide a historical overview illustrating the evolution of carrier detection and prenatal diagnosis of HA over the last 4 decades (Figure 1). In addition, we discuss the relatively rare phenomenon of skewed X chromosome inactivation (XCI) in symptomatic HA carriers, with special emphasis on the crucial importance of further genetic analysis aimed at identifying additional deleterious mutations potentially involved in XCI skewing. Finally, we present future directions toward promising clinical applications in the field of genetic diagnosis of hemophilia.

## 2. Historical Overview of HA Carrier Detection and Prenatal Diagnosis

### 2.1. Carrier Detection

In the late 1970s and early 1980s, carrier detection of HA was based on meticulous pedigree analysis aimed to identify potential carriers, combined with phenotype analysis using coagulation studies for confirmation or exclusion of carriership. The phenotypic method of carrier detection involves measurements of Factor VIII coagulant activity (FVIIIC) and von Willebrand factor (VWF) antigen (VWF Ag) levels, followed by statistical analysis based on the FVIIIC/VWF Ag ratio, which is reduced in HA carriers [1,2]. However, both FVIIIC and VWF Ag levels are significantly affected by various factors, such as age, ABO type [7], as well as pregnancy [8], all of which might influence the phenotypic characteristics of HA carriers, possibly resulting in misdiagnosis of HA carrier status. Attempts to enhance the accuracy of carrier detection by phenotypic methods included multiple tests in separate samples of the potential carrier [1,2], and analysis of FVIIIC/VWF Ag ratio prior to and following DDAVP administration. DDAVP was demonstrated to induce a similar increase in VWF Ag levels in both HA carriers and normal women, with a much smaller increase in FVIIIC levels in HA carriers compared to normal women [9].

Characterization of the F8 gene in the mid-1980s launched a new era of HA genetic diagnosis by molecular biology techniques [10]. The F8 gene is extremely large and complex, comprised of 26 exons and spanning 186 kb. In the late 1980s and early 1990s, genetic studies of HA families primarily relied on indirect linkage analysis of polymorphic markers (restriction fragment length polymorphisms (RFLP), currently referred to as single nucleotide polymorphisms (SNPs)) situated within or in close proximity to the F8 gene. RFLP/SNP analysis, originally conducted by the extremely time-consuming and labor-intensive Southern blot method [11], has been greatly simplified by the development of PCR techniques in the late 1980s [12].

Extensive studies searching for polymorphic markers in the F8 gene revealed that the F8 gene is surprisingly hypo-polymorphic, with only four intragenic RFLPs/SNPs found in such a large gene: BclI RFLP/SNP in intron 18, XbaI RFLP/SNP in intron 22, HindIII RFLP/SNP in intron 19, and BglI RFLP/SNP in intron 25 [13,14]. The prerequisite for the usefulness of a certain polymorphic marker in carrier detection and/or prenatal diagnosis is a high rate of heterozygosity for that marker. Unfortunately, the usefulness of the aforementioned bi-allelic RFLPs/SNPs has been greatly limited by low rates of heterozygosity and linkage disequilibrium [15]. The discovery of highly polymorphic and multi-allelic short tandem repeats (STRs) in intron 13 [16] and intron 22 [17] of the F8 gene has significantly enhanced the rates of heterozygosity, resulting in improved efficacy of linkage studies in HA families. Further studies, enabled by the progress of the Human Genome Project, have revealed additional STRs located in close proximity to the F8 gene, thus expanding the reservoir of potentially informative polymorphic markers and enabling the construction of a specific hemophilic haplotype useful for genetic analysis of each HA family [18].

Although highly precise carrier detection can be achieved by linkage studies in families with a history of HA, identification of the hemophilic haplotype in a female relative of a sporadic HA patient does not necessarily indicate hemophilia carriership. In such cases, detection of the causative F8 gene mutation is crucial for accurate and unequivocal carrier detection.

Significant advances in molecular biology techniques have greatly facilitated the identification of causative F8 gene mutations in HA patients. Early mutation analyses based on PCR amplification of F8 promoter, coding regions, and splice junctions revealed causative mutations in almost all patients with mild and moderate HA, but in only half of patients with severe HA [19]. Further studies based on PCR amplification of reverse transcribed mRNA demonstrated that about half of severe HA patients exhibit abnormal mRNA sequences in the region of exons 22–23, i.e., absence of PCR amplification across the boundary between exons 22 and 23, indicating absence of the normal joining of exon 22 to 23 in the factor VIII mRNA [20]. These important findings have subsequently led to the identification and detailed characterization of intron 22 inversions accounting for about 45% of severe HA cases [21]. At present, the common intron 22 inversions and the less common recurrent intron 1 inversion [22], together accounting for almost 50% of severe HA cases, can be precisely identified by relatively simple PCR assays [22,23]. The causative F8 mutations include missense and nonsense mutations, small insertions/duplications/deletions, large duplications/deletions [Factor VIII Gene (F8) Variant Database; https://f8-db.eahad.org/reference.html.php (accessed on 1 July 2023)], as well as deep intronic mutations potentially creating alternative splicing sites [24,25]. Identification of this plethora of pathogenic variants is accomplished by either Sanger sequencing of each of the 26 exons of the F8 gene or by application of NGS methods. Definite identification of the F8 mutation enables accurate assessment of HA carrier status in all the relevant female relatives of the diagnosed HA patient, as well as prenatal diagnosis of HA for HA carriers, depending on their individual reproductive choices.

### 2.2. Prenatal Diagnosis

The aim of prenatal diagnosis is either to prevent the birth of a child with HA or to determine specific precautions required for the prevention of potential obstetrical complications during pregnancy and delivery of a child diagnosed with HA.

In the early period of phenotypic diagnosis of HA, prenatal diagnosis offered to established HA carriers was based on fetal sexing by amniocentesis at week 16 of pregnancy, followed by measurement of FVIIIC levels in fetal blood samples obtained from male fetuses at week 20 [4]. However, this procedure could be offered only in a very limited number of highly specialized medical centers, and even in those centers, the estimated risk of fetal death was 2–5% [4].

The introduction of the chorionic villus sampling (CVS) technique in the early 1990s has enabled early prenatal diagnosis of HA during weeks 11–12 of pregnancy [26,27]. Examination of fetal DNA extracted from the chorionic villus tissue for the familial hemophilic haplotype or the causative F8 mutation, combined with analysis of multiple microsatellite markers aimed at eliminating the risk of misdiagnosis due to maternal contamination of fetal DNA [28], enables definite diagnosis of HA during the first trimester of pregnancy.

The development of highly sensitive PCR methods assisting in the identification of fetal DNA in maternal blood has enabled early determination of fetal sex by examination of Y chromosome sequences at weeks 5–10 of pregnancy [29,30]. Early fetal sexing may help HA carriers to avoid unnecessary CVS procedures in pregnancies with female fetuses, thus eliminating the risk of miscarriage associated with the CVS procedure.

The attitude towards prenatal diagnosis of hemophilia significantly varies between different world regions. In view of the continuous advances and increasing availability of diverse therapeutic options in developed countries, hemophilia patients are now capable of leading a normal life, with life expectancy equivalent to that observed in the general population. Therefore, acceptance of prenatal diagnosis has been gradually decreasing in these countries. However, prevention of severe hemophilia is crucial in developing countries, where patients rarely survive beyond childhood in view of inadequate therapy due to the extremely limited availability of therapeutic products. Beyond national public health policies and the availability of hemophilia prevention options in various world regions, personal attitudes to living with hemophilia have a significant impact on the carriers’ choices regarding giving birth to an affected child. Personal beliefs, as well as religious and cultural constraints, greatly contribute to these attitudes, which must always be carefully considered in the setting of genetic counseling.

In the Israeli National Hemophilia Center, the approach of early fetal sexing at week 7–8 of pregnancy, followed by CVS for prenatal diagnosis of HA in pregnancies with male fetuses, has evolved into the standard of care successfully implemented in the National Hemophilia Prevention Program over the last 2 decades. In Poland, prenatal testing for hemophilia is less widespread and hemophilia is not considered an indication for pregnancy termination in view of the rapidly improving management of the disease. A study comparing the attitudes towards prenatal diagnosis of hemophilia and termination of pregnancy in Iran and Italy demonstrated a much higher rate of acceptance of abortion among Iranian participants (58.2%) as compared to Italian participants (16.7%). The significantly greater rate of acceptability of abortion in the Iranian population may be attributed to differences in the quality of patient care in the two countries [31].

### 2.3. Preimplantation Genetic Diagnosis (PGD)

Preimplantation genetic diagnosis (PGD) involves in vitro fertilization (IVF) in conjunction with genetic diagnosis of HA in the embryo, followed by transfer of an embryo free of HA to the HA carrier. Single cells biopsied from cultured embryos at the stage of 6 to 8 cells are examined for the hemophilic haplotype using multi-allelic STR markers within and in close proximity to the F8 gene. Predetermined informative STR markers are co-amplified with AMELX/Y indel polymorphism for sex determination in a single-tube PCR reaction [32,33,34,35]. At least two informative STR markers, preferably located upstream and downstream of the F8 gene, must be used to avoid the risk of misdiagnosis due to recombination between the marker and the F8 mutation. The first PGD for HA prevention was carried out in Colombia in 1994 [36]. Over the last 3 decades, PGD programs have been successfully implemented in numerous medical centers worldwide, and this approach has gradually become the procedure of choice for HA carriers who reject the option of prenatal diagnosis and termination of pregnancy in case of an affected fetus [37].

### 2.4. Genetic Analysis of Symptomatic HA Carriers

Heterozygous female HA carriers are typically asymptomatic because their FVIII levels correspond to approximately half of the concentration found in healthy individuals, which is generally sufficient for normal hemostasis [7]. However, in rare cases, HA carriers may exhibit symptoms of mild to severe hemophilia.

The genetic mechanisms underlying the manifestation of HA phenotype in HA carriers include homozygosity or compound heterozygosity for F8 gene mutations [38,39], Turner syndrome [39,40,41], misdiagnosed VWD type 2N [39,42], or abnormally skewed inactivation of the normal X chromosome [43].

Studies investigating the genetic causes of HA phenotype in HA carriers indicate that skewed XCI accounts for the majority of cases [44]. XCI is a normal process correcting the disparity in gene copy numbers between males and females. The potential excess of gene expression from both X chromosomes in females is eliminated by the inactivation of one of the two X chromosomes in each somatic cell, resulting in the expression of only one allele at the vast majority of X-encoded loci. XCI is generally a random process, resulting in approximately equal expression of both maternal and paternal X chromosome genes. Thus, a heterozygous female HA carrier with random XCI usually exhibits an adequate expression of about 50% of normal FVIII levels. However, on rare occasions, non-random XCI may occur, resulting in the inactivation of the normal X chromosome in a symptomatic female carrier [38,45]. Of note, a female carrier of HA and moyamoya (SHAM) syndrome, caused by Xq28 deletion encompassing F8 and the BRCC3 familial moyamoya gene, was reported to exhibit both HA and cardiovascular morbidity symptoms due to skewed inactivation of the X chromosome without the Xq28 deletion [46].

Although extremely skewed XCI can be accidental, such a rare phenomenon may be mediated by a mechanism of selection against a deleterious mutation in a gene located on the silenced X chromosome bearing the intact F8 gene copy.

Table 1 presents the results of studies evaluating X chromosome pathogenic variants potentially causing skewed XCI in symptomatic HA carriers.

Pavlova et al. [38] (Table 1, Patient 1) described a severely symptomatic HA carrier carrying a frameshift mutation in the F8 gene (c.6872 delCT) combined with a nonsense mutation (Arg110*) in the RSK2 gene (NM_004586.3; currently named RPS6KA3 (ribosomal protein S6 kinase A3)). The patient exhibited complete inactivation of her X chromosome bearing the intact F8 gene and the mutant RSK2 gene. The patient had two brothers, hemizygous for the RSK2 mutation, who were diagnosed with Coffin–Lowry syndrome, a rare genetic disorder characterized by intellectual disability. Notably, skewed inactivation of the X chromosome bearing the mutant RSK2 mutation has been observed in carriers of Coffin-Lowry syndrome, supporting the authors’ hypothesis that selection against RSK2 mutant cells may have resulted in skewed XCI in the female HA patient described above [38].

Based on X chromosome-targeted WES analysis, deleterious mutations in genes located on the X-chromosome bearing the intact F8 gene copy have been implicated in XCI skewing, resulting in manifestation of moderate to severe HA phenotype in 3 unrelated HA carriers treated and followed by the Israeli National Hemophilia Center [patients 2–4, [47]], and in 3 unrelated HA carriers treated and followed by hemophilia centers in Poland [patients 5–7, [39]].

Patient 2 (Table 1), a heterozygous carrier of a splice site mutation in the F8 gene (IVS 5 + 2 T > G) presenting with severe HA phenotype (FVIII < 1%), exhibited skewed inactivation of her non-hemophilic X chromosome, which was associated with a frameshift mutation (NM_000291.4; c.1061_1062delCT; p.A354fs*4) in the phosphoglycerate kinase 1 (PGK1) gene encoding the PGK1 enzyme, which plays a key role in the glycolysis pathway. PGK1 deficiency has been reported to be associated with chronic anemia, exercise-intolerant myopathy, muscle weakness, cramping, myalgia, myoglobinuria, and intellectual disability [48].

Patient 3 (Table 1), a heterozygous carrier of F8 intron 22 inversion presenting with moderate HA phenotype (FVIII 4%), exhibited skewed inactivation of her non-hemophilic X chromosome, which was associated with a nonsense mutation in the NF-kappa B activating protein (NKAP) gene (NM_024528; c.175C > T; p. Q59*) encoding the NKAP protein. NKAP is involved in the activation of the ubiquitous transcription factor NF-Kappa B. NKAP knockout has been reported to be perinatally lethal in mice [49]. Deleterious missense mutations in the NKAP gene have been associated with developmental delay, hypotonia, joint contractures, behavioral abnormalities, Marfanoid habitus, and scoliosis [50].

Patient 4 (Table 1), a heterozygous carrier of F8 intron 22 inversion presenting with moderate HA phenotype (FVIII 3%), exhibited skewed inactivation of her non-hemophilic X chromosome, which was associated with a missense mutation in the synaptotagmin-like protein 4 (SYTL4) gene (NM_001129896; c.1655A > C; p.K552T) encoding the SYTL4 protein. SYTL4 is involved in intracellular membrane trafficking via interaction with Rab GTPases, and plays an important role in neuronal system development. SYTL4 mutations have been implicated in neurological and psychological diseases [51,52].

Patient 5 (Table 1), a heterozygous carrier of an F8 mutation causing mild HA (c.6929C > T; p.Thr2310Ile), presenting with mild HA phenotype (FVIII 24.5%) due to skewed XCI, was diagnosed as a carrier of a missense mutation in the HS6ST2 gene (NM_147175.4; c.349C > T; p.Arg117Trp) encoding heparan sulfate 6-O-sulfotransferase 2 (HS6ST2). HS6ST2 is involved in the structural modification of HSPGs, which is required for their interactions with a variety of proteins. These interactions are implicated in diverse cellular processes, including proliferation, differentiation, adhesion, and migration [53]. A detrimental HS6ST2 gene variant has been reported to be associated with X-linked intellectual disability in two male twins [54].

Patient 6 (Table 1), a heterozygous carrier of an F8 mutation causing severe HA (c.1271 + 1G > T), presenting with moderate HA phenotype (FVIII 3%) due to skewed XCI, was diagnosed as a carrier of a missense mutation in the HCFC1 gene (NM_005334.3; c.5752G > A; p.Gly1918Ser) encoding Host Cell Factor C1 (HCFC1). HCFC1, a co-regulator of the zinc-finger transcription factor THAP11, has been demonstrated to regulate a variety of processes, including cell cycle, proliferation, and transcription via interaction with various proteins [55,56]. Yu et al. have identified five hemizygous pathogenic missense mutations in the HCFC1 gene in 14 unrelated males with X-linked intellectual developmental disorder [57].

Patient 7 (Table 1), a heterozygous carrier of an F8 mutation causing severe HA (c.1812G > C; p.Trp604Cys), presenting with severe HA (FVIII < 1%) due to skewed XCI, was diagnosed as a carrier of a frameshift mutation in the RLIM gene (NM_016120.4; c.25_28del; p.Lys9GlufsTer27) encoding the ubiquitin protein ligase RING zinc finger protein 12 (RNF12). RNF12 has been implicated in the regulation of numerous protein levels via proteasomal degradation [58,59,60,61]. Furthermore, RNF12 plays crucial roles in neuronal development and function [62], and in normal X inactivation in mice [59]. Tonne et al. [63] have reported a three-generation Norwegian family with X-linked intellectual disability (XLID) syndrome segregating with a missense mutation in the RLIM gene. The authors have also indicated that all female carriers of the RLIM mutation exhibited a completely skewed XCI pattern, suggesting that RLIM may be essential for normal X-chromosome inactivation in humans as well.

## 3. Mosaicism in Mothers of Sporadic HA Patients

Approximately 30% of HA patients are sporadic cases with no family history of HA.

In the vast majority of sporadic HA cases, the mother of the index case is a carrier of the causative F8 mutation, which is detectable in her white blood cell DNA. A mother who does not exhibit the causative F8 mutation in her white blood cell DNA may be either a non-carrier (i.e., the causative F8 mutation has occurred in the first HA patient during embryonic development), or a mosaic carrying the mutation in some of her tissues, which may include her germline cells (i.e., germline or gonadal mosaicism). In such a scenario, the apparently non-carrier mother may be at substantial risk of giving birth to additional HA patients.

Previous studies suggest that the occurrence of mosaicism may be associated with the type of mutation causing HA. A large international study by Antonarakis et al. [64] conducted in 2093 patients with intron 22 inversions, the most common genetic defect causing HA, demonstrated that 96% of the mothers of sporadic HA patients were carriers of the causative intron 22 inversion. Examination of the mutation origin revealed that de novo inversions occurred almost exclusively (in 98% of cases) in the germ cells of the patient’s healthy maternal grandfather [64]. A possible explanation for the much higher rate of intron 22 inversion occurrence in male vs. female germ cells may be related to the fact that the Xq arm is unpaired during sperm cell meiosis, thus enabling flipping of the X-chromosome tip, which may be inhibited by the homologous pairing of the X chromosomes during oocyte meiosis. Similarly to intron 22 inversions, point mutations also show a 5–10-fold higher mutation rate in male germ cells. In contrast, large gene rearrangements, such as deletions and duplications, are hypothesized to originate predominantly in female germ cells [65].

In a study of a large Chinese cohort that included 257 families with sporadic HA cases, Lu et al. demonstrated mosaicism in 22.5% of mothers determined to be non-carriers by analysis of their white blood cell DNA [66]. A similar rate of mosaicism (20%) was reported by Martensson et al. in a Swedish cohort of 45 families with sporadic HA cases [67].

In view of the relatively high incidence of mosaicism among mothers of sporadic HA patients, comprehensive genetic analysis aimed at detecting mosaicism, using highly sensitive methods such as quantitative PCR or NGS in DNA samples extracted from various tissues, such as buccal cells, hair follicles, and urothelial cells must be conducted in order to enable accurate assessment of the risk of HA recurrence in the future offspring. In some cases, analysis of ovarian cells for the causative F8 mutation may be necessary [68].

## 4. Current Challenges and Future Directions in Genetic Analysis of HA

### 4.1. Failure to Detect Causative F8 Mutations by Conventional Genetic Techniques

The conventional approach currently used for molecular diagnosis of HA is based on screening for the common intron 22 and intron 1 inversions, sequencing of all the 26 F8 exons, splice junction regions, promoter and 3′-untranslated region, and analysis of copy number variation (CNV). Despite such a comprehensive analysis, the causative F8 mutation remains unidentified in 1–5% of HA patients. In these cases, the causative F8 mutation might be a deep intronic variant creating a de novo acceptor splice site or donor splice site. Several groups have used whole F8 gene sequencing by next-generation sequencing (NGS) in order to identify the pathogenic mutation in HA patients who remained undiagnosed following application of the conventional diagnostic approach. Using whole F8 gene sequencing combined with splicing functional analyses, Dericquebourg et al. [69] have recently identified pathogenic F8 deep intronic variants as causative mutations in 67% (33 of 49) of HA patients unresolved by conventional genetic methods. Earlier studies based on whole F8 gene sequencing have reported the identification of pathogenic deep intronic variants in 47% [70] and 60% [71] of genetically unresolved HA patients.

The aforementioned studies emphasize the importance of whole F8 gene sequencing combined with splicing functional analyses for elucidation of the disease-causing mutation in genetically undiagnosed HA patients. However, such comprehensive studies are not yet broadly available in routine clinical practice, especially in developing countries. Therefore, in hemophilia centers with no access to NGS technology, carrier detection in female relatives of a HA patient with no identified F8 mutation should be based on comprehensive genetic linkage studies combined with analysis of FVIIIC and VWF Ag levels.

### 4.2. Risk of Fetal Loss due to Invasive Prenatal Diagnosis

Currently, fetal genotyping involves invasive procedures such as chorionic villus sampling, amniocentesis, or fetal blood sampling. All these procedures are associated with a substantial risk of fetal loss.

The discovery of cell-free fetal DNA in maternal plasma [72] has led to the development of non-invasive prenatal testing (NIPT) techniques, with successful clinical implementation in the diagnosis of fetal aneuploidies and monogenic diseases of paternal origin [73]. Analysis of maternal plasma for Y chromosome sequences is already being used for fetal sex determination as the first step in prenatal diagnosis of HA. However, the detection of a male fetus requires the HA carrier to undergo an invasive procedure such as CVS or amniocentesis aimed at obtaining fetal tissue for HA diagnosis. Several studies have reported that digital PCR-based methods can be successfully applied for non-invasive prenatal diagnosis of HA. Hudecova et al. has demonstrated the correct determination of fetal hemophilia status by droplet digital PCR analysis in maternal plasma samples of 15 hemophilia carriers [74]. Tsui et al. have successfully applied digital PCR and relative mutation dosage analysis for non-invasive hemophilia diagnosis in 12 maternal plasma samples [75]. Unfortunately, these promising results have not yet been translated into clinical practice. Further studies are required for the validation of digital PCR methods in non-invasive prenatal diagnosis of recessive monogenic diseases of maternal origin.

### 4.3. Symptomatic HA Carriers: Detection of Additional Deleterious Mutations and Their Impact on Reproductive Choices

The presence of additional deleterious X-linked mutations in symptomatic HA carriers may influence their reproductive choices [76], and, therefore, must be carefully considered by geneticists designing the prenatal diagnosis approach for such carriers. In view of the significant association of additional deleterious mutations, potentially causing XCI skewing (Table 1), with severe X-linked developmental delay and intellectual disability, IVF combined with PGD should be offered as the procedure of choice to eliminate the risk of birth of a male child affected with either severe HA or X-linked intellectual disability. A female embryo carrying one of the mutant genes, based on considerations of pathogenicity associated with each of them, should be selected for transfer to a symptomatic HA carrier diagnosed with an additional X-linked deleterious mutation. This approach has been successfully applied in family planning for patient 2 (Table 1), a symptomatic HA carrier with a deleterious PGK1 gene mutation. She chose the option of a female embryo carrying the mutant PGK1 gene (but not the mutant F8 gene), who was subsequently diagnosed with skewed XCI of the X chromosome bearing the mutant PGK1 gene [46].

## 5. Conclusions

Highly accurate molecular biology techniques enabling precise diagnosis of the HA-causing mutation are not yet widely available in routine clinical practice. Expanding access to mutation analysis is especially relevant for developing countries, where carrier detection and prenatal diagnosis of HA are crucially important in view of the low availability of therapeutic products; thus, worldwide collaboration projects such as My Life, Our Future initiative [77], successfully implemented in the US for genetic analysis of 3000 hemophilia patients, are required to fulfill the unmet need of precise molecular diagnosis of HA in hemophilia centers with no access to advanced molecular biology techniques.

Detection of mosaicism in mothers of sporadic HA patients who appear to be non-carriers (i.e., not exhibiting the causative *F8* mutation in DNA extracted from their blood cells), based on analysis of multiple tissues (such as hair follicles, buccal cells, and urine samples) using highly sensitive technologies, is relatively expensive and time-consuming. However, the identification of mosaicism is highly informative for both the suspected carrier and genetic counselors. Detection of the causative *F8* mutation in any of the tissues examined may suggest that the mutation is present in the suspected carrier’s ovaries as well, and therefore, the risk of HA recurrence should be discussed and managed accordingly.

Finally, symptomatic hemophilia carriers require extensive genetic analysis beyond their HA carrier status. The ultra-rare phenomenon of manifestation of hemophilia in a female, as discussed above, may indicate carriership of severe X-linked disease on the non-hemophilic X-chromosome. Therefore, we recommend whole exome sequencing with X chromosome-targeted bioinformatic analysis for all symptomatic hemophilia carriers with skewed XCI. A hemophilia carrier diagnosed with an additional X-linked deleterious mutation should receive comprehensive genetic counseling with the aim of appropriately informing and guiding her reproductive decisions.

## Figures and Tables

**Table 1:** Additional deleterious X-linked mutations were identified in symptomatic HA carriers.

Symptomatic HA Carrier (Reference)	F8 Mutation	Additional Mutant Gene Potentially Involved in XCI Skewing	Degree of XCI Skewing	HA Severity
1 [38]	c.6872 delCT Thr2272fs	*RSK2* (Arg110*) Protein: Ribosomal S6 Kinase 2	Severe	Severe
2 [47]	Intron 22 inversion	*NKAP * (NM_024528; c.175C > T; p. Q59*) Protein: NF kappa B Activating Protein	Severe	Moderate
3 [47]	Intron 22 inversion	*SYTL4* (NM_001129896; c.1655A > C; p.K552T) Protein: Synaptotagmin Like 4	Severe	Moderate
4 [47]	IVS5 + 2 T > G	*PGK1 * (NM_000291.4; c.1061_1062delCT; p.A354fs*4) Protein: Phosphoglycerate Kinase 1	Severe	Severe
5 [39]	c.6929C > T; p.Thr2310Ile	*HS6ST2* (NM_147175.4; c.349C > T; p.Arg117Trp) Protein: Heparan Sulfate 6-O-Sulfotransferase 2	Severe	Mild
6 [39]	c.1271 + 1G > T	*HCFC1* (NM_005334.3; c.5752G > A; p.Gly1918Ser) Protein: Host Cell Factor C1	Severe	Moderate
7 [39]	c.1812G > C; p.Trp604Cys	*RLIM* (NM_016120.4; c.25_28del; p.Lys9GlufsTer27) Protein: Ring Finger, LIM Domain Interacting	Severe	Severe

## Data Availability

Not applicable.
